# Impact of Empirical and Definitive Antibiotics on Pediatric Febrile Urinary Tract Infection Caused by ESBL-Producing Enterobacterales

**DOI:** 10.3390/pathogens14111103

**Published:** 2025-10-29

**Authors:** Jin Lee, Yejin Kim, Ye Ji Kim, Seung Beom Han, Jin-Soon Suh, Soo Young Lee, Jong-Hyun Kim

**Affiliations:** Department of Pediatrics, College of Medicine, The Catholic University of Korea, Seoul 06591, Republic of Korea; pedleejin@catholic.ac.kr (J.L.); yejin1215@gmail.com (Y.K.); jenniferyejikim@gmail.com (Y.J.K.); rebekahjs@catholic.ac.kr (J.-S.S.); sylee@catholic.ac.kr (S.Y.L.); jh00mn@catholic.ac.kr (J.-H.K.)

**Keywords:** urinary tract infections, extended-spectrum β-lactamase, antibiotics, children

## Abstract

Although the clinical impact of empirical antibiotic susceptibility on urinary tract infection (UTI) caused by extended-spectrum β-lactamase-producing Enterobacterales (ESBL-PEs) is limited, the role of definitive antibiotics remains unclear. This study evaluated therapeutic outcomes and recurrence in children with febrile UTI caused by ESBL-PE, focusing on pathogen susceptibility to empirical and definitive antibiotics. Medical records of 376 UTI episodes caused by ESBL-PE in children aged <10 years were retrospectively reviewed, and 319 were analyzed. Episodes were classified as empirical-susceptible (*n* = 144) and empirical-non-susceptible (*n* = 175) groups, and further categorized into four groups based on definitive antibiotic susceptibility and treatment duration. Clinical outcomes were comparable between empirical-susceptible and empirical-non-susceptible groups. Among the four definitive-therapy groups, early clinical response did not differ significantly; however, UTI recurrence showed an increasing trend from 1.0% in those receiving empirical and definitive antibiotics to which pathogens were susceptible for ≥7 days to 6.3% in those receiving empirical antibiotics to which pathogens were non-susceptible and not receiving antibiotics to which pathogens were susceptible ≥7 days (*p* = 0.045). Despite favorable early response to empirical non-susceptible antibiotics, switching to susceptible antibiotics and maintaining therapy for ≥7 days is recommended for children with ESBL-PE UTIs.

## 1. Introduction

Urinary tract infection (UTI) is one of the most common bacterial infections in children, with a prevalence of approximately 7% [1]. Enterobacterales, particularly *Escherichia coli* and *Klebsiella pneumoniae*, cause most cases of UTIs, and therefore, β-lactam antibiotics, such as penicillins and cephalosporins, are recommended by the American Academy of Pediatrics (AAP) as empirical therapy for children with suspected UTI, based on local susceptibility patterns [2]. However, these agents are regarded as ineffective against extended-spectrum β-lactamase (ESBL)-producing organisms, the prevalence of which has been increasing among UTI-causing Enterobacterales [3,4]. In addition, ESBL-producing Enterobacterales (ESBL-PEs) are often resistant to other classes of antibiotics beyond β-lactams [4]. Therefore, carbapenems are generally recommended for infections caused by ESBL-PEs, except for certain mild cases [5]; however, they cannot be administered orally, and their use for largely non-life-threatening infections such as UTI may be inappropriate. Furthermore, the growing prevalence of carbapenem-resistant Enterobacterales as a consequence of increased carbapenem use has emerged as an important public health concern [6]. Although carbapenem resistance currently has less clinical impact in children than in adults [7], restricted use of carbapenems should be emphasized in children, given the limited therapeutic options for infections caused by carbapenem-resistant microorganisms. Recently developed β-lactam and β-lactam–β-lactamase inhibitor combination agents, such as cefiderocol, ceftazidime–avibactam, meropenem–vaborbactam, and imipenem–cilastatin–relebactam, are promising against ESBL-PE infections; however, they should be reserved for more complicated infections [4].

A previous history of UTI, recent antibiotic use or hospitalization, and underlying urogenital abnormalities, including vesicoureteral reflux (VUR), are established risk factors for UTI caused by ESBL-PEs [3,8,9]. However, in real-life clinical settings, it is often infeasible to provide differentiated empirical antibiotic therapy for the relatively small subset of patients suspected of having ESBL-PE infections, which account for approximately 14% of pediatric UTI cases [3]. Encouragingly, some non-carbapenem antibiotics, including β-lactam–β-lactamase inhibitor combinations, aminoglycosides, trimethoprim-sulfamethoxazole (TMP-SMX), and fluoroquinolones, have been reported to be effective in UTIs caused by ESBL-PEs [4]. Nevertheless, most previous studies excluded third-generation cephalosporins from their analyses, despite these agents being the most commonly used antibiotics for UTI. Furthermore, only a few studies with small sample sizes have evaluated therapeutic effects and outcomes according to definitive antibiotics rather than empirical therapy, without consideration for the duration of definitive antibiotic therapy [10,11,12]. In children, research on antibiotic use in the context of ESBL-PE infections remains more limited than in adults [8,12,13,14,15].

This study aimed to evaluate therapeutic outcomes and recurrence in children with febrile UTI caused by ESBL-PEs, with a focus on pathogen susceptibility to both empirical and definitive antibiotics. The findings may provide valuable evidence to guide appropriate antibiotic strategies for pediatric UTIs caused by ESBL-PEs.

## 2. Materials and Methods

### 2.1. Patients and Study Design

Episodes of UTI caused by ESBL-PEs, diagnosed in children younger than 10 years between January 2010 and December 2019 at three university-affiliated hospitals of the Catholic Medical Center in Korea, were included in this study. The participating hospitals were Seoul St. Mary’s Hospital in Seoul, the capital city of Korea, and Bucheon St. Mary’s Hospital and St. Vincent’s Hospital, both located in the metropolitan area. UTI episodes were excluded if the child remained afebrile throughout the illness or if the infection was hospital-acquired. De-identified medical records of the included children were retrospectively reviewed using data extracted from the Clinical Data Warehouse of the Catholic Medical Center. Demographic data, including sex and age, and clinical data, including previous history of UTI; underlying medical conditions including urogenital abnormalities; prior hospitalization and antibiotic therapy within the preceding 3 months; durations of hospitalization, fever, and antibiotic therapy; concurrent diagnoses at the time of UTI; and the types of administered antibiotics were collected. Recurrence of UTI within 3 months after the index episode was assessed, and information on subsequently performed urological interventions was also collected. Imaging study results, including renal and bladder ultrasonography, 99mTc-dimercaptosuccinic acid scans, and voiding cystourethrography performed within 3 months before or after the diagnosis of UTI, were reviewed. Causative pathogens were identified based on urine culture, and their antibiotic susceptibility test results were analyzed.

The included UTI episodes were first divided into two groups based on the susceptibility of the identified ESBL-PE to empirical antibiotics: the empirical-susceptible group and the empirical-non-susceptible groups. Subsequently, the episodes were further categorized into four groups according to susceptibility to both empirical and definitive antibiotics, as well as the duration of definitive antibiotic therapy.

- Group 1: Empirical and definitive therapy with antibiotics to which the isolated pathogens were susceptible, continued for ≥7 days.
- Group 2: Empirical therapy started with non-susceptible antibiotics, but switched to susceptible definitive antibiotics, administered for ≥7 days.
- Group 3: Empirical therapy started with susceptible antibiotics, but switched to non-susceptible definitive antibiotics within 7 days.
- Group 4: Empirical therapy started with non-susceptible antibiotics, and susceptible antibiotics were not administered for ≥7 days during the entire treatment course, regardless of the definitive regimen.

Considering that the AAP guideline for pediatric febrile UTI recommends 7–14 days of antibiotic therapy [2], the minimum duration of 7 days was selected as the threshold for adequate susceptible antibiotic therapy.

The collected clinical data were compared between the empirical-susceptible and empirical-non-susceptible groups. Additional comparisons among Groups 1–4 were conducted to evaluate the effects of antibiotic susceptibility and treatment duration on clinical outcomes. This study was approved by the Institutional Review Board of Bucheon St. Mary’s Hospital with a waiver of acquiring informed consent (approval No.: HC25WIDI0066 and approval date: 2 September 2025).

### 2.2. Microbiological Testing

Urine samples were collected using a sterile urine collection bag applied over the perineum after skin disinfection in non-toilet-trained children, or as clean-catch midstream urine in toilet-trained children. Urine cultures were performed using conventional culture techniques. Bacterial species identification, antibiotic susceptibility testing, and ESBL detection were performed using the Vitek^®^2 automated system (bioMérieux, Marcy l’Etoile, France), and no additional confirmatory testing for ESBL was conducted. Antibiotic susceptibility was interpreted according to the Clinical and Laboratory Standards Institute criteria, as revised during the study period [16]. Intermediate and resistance results were classified as non-susceptible. Susceptibility to cephalosporins was assigned according to the minimum inhibitory concentration criteria, regardless of ESBL positivity. For third-generation cephalosporins, cephamycins, fluoroquinolones, and carbapenems, when any of the tested antibiotics in the antibiotic class was non-susceptible, the entire class was designated as non-susceptible. Susceptibility testing was performed for cefotaxime or ceftazidime among third-generation cephalosporins; for cefoxitin or cefotetan among cephamycins; for ciprofloxacin, levofloxacin, or norfloxacin among fluoroquinolones; and for imipenem, meropenem, or ertapenem among carbapenems, depending on the hospital and study period.

### 2.3. Definitions

The diagnosis of UTI was made when both pyuria and bacteriuria were present in febrile children. Pyuria was defined as >10 white blood cells/μL, measured by automated urine flow cytometry in unspun urine or by microscopic examination of centrifuged urine sediment. Bacteriuria was defined as growth of ≥100,000 colony-forming units/mL of a single pathogen in urine culture. UTI episodes diagnosed ≥ 48 h after admission or <48 h after discharge from a previous hospitalization were considered hospital-acquired, and were excluded. Recurrent UTI was defined as a new episode diagnosed between 2 weeks and 3 months after completion of treatment for a previous UTI, with isolation of the same ESBL-PE strain (same species showing the same antibiotic-susceptibility pattern) in urine culture. Fever was defined as a body temperature of ≥38 °C measured by a tympanic membrane thermometer in hospital or by any types of thermometers at home. Defervescence was defined as a body temperature <38 °C sustained for ≥48 h after the last fever. Total fever duration was defined as the number of days from the first to the last day of fever. Fever duration after the initiation of antibiotic therapy was calculated in hours, from the start of antibiotic treatment to the last fever before defervescence. Empirical antibiotics were defined as the initial agents administered during the UTI episode, whereas definitive antibiotics were defined as the first agents given according to the antibiotic susceptibility test results. If empirical antibiotics were continued without change after the susceptibility report, definitive antibiotics were considered identical to empirical regimen. Urological interventions included antibiotic prophylaxis and urological surgical procedures performed up to the later of the two dates: the patient’s last visit to the study hospital or 31 December 2024.

### 2.4. Statistical Analysis

Comparisons between the empirical-susceptible and empirical-non-susceptible groups were performed using the Mann–Whitney U test for continuous variables, as none followed a normal distribution according to Shapiro–Wilk test. Categorical variables were analyzed with the chi-square test. For comparisons among Groups 1–4, the Kruskal–Wallis test was used for continuous variables, followed by post hoc analysis with Bonferroni adjustment. Categorical variables were compared using the linear-by-linear association test to evaluate linear trends across ordinal categories, reflecting the effects of antibiotic susceptibility and treatment duration. All statistical analyses were conducted using the R software program (v4.3.3, R Core Team 2024, R Foundation for Statistical Computing, Vienna, Austria), and cases with missing data were excluded from the corresponding analyses. Statistical significance was set at *p*-value < 0.05.

## 3. Results

### 3.1. Clinical Characteristics of Children with UTI Caused by ESBL-Producing Enterobacterales

During the study period, 376 episodes of UTI caused by ESBL-PE were identified in 352 children. Two children experienced four episodes each, and eighteen experienced two episodes each. Of all episodes, 366 (97.3%) were caused by *E. coli*, and 10 (2.7%) by *K. pneumoniae* (Table 1). The median age of the included children was 5 months (range 0–104), with 234 (62.2%) younger than 6 months. Males accounted for 249 (66.2%) episodes, outnumbering females (33.8%, *n* = 127). Most (85.9%, *n* = 323) episodes represented first occurrences, and hospitalization was required in 328 (87.2%) episodes, with a median duration of 7 days (range 1–35). Previous hospitalization and antibiotic use within 3 months prior to UTI diagnosis were identified in 63 (16.8) and 77 (20.5%) episodes, respectively. Urogenital anomalies were identified in 56 (14.9%) episodes, with VUR being the most common (Table 1). None of the children had a prior history of urological interventions. Other underlying medical conditions were identified in 11 (2.9%) episodes: hemodynamically stable congenital heart disease in 7, and congenital megacolon, sensorineural hearing loss, cleft lip and palate, and acute leukemia in 1 episode each. Concurrent diagnoses were present in 50 (13.3%) episodes, most commonly upper respiratory infection (Table 1). Bacteremia occurred in six (1.6%) episodes, all of which were caused by the same ESBL-producing *E. coli*, identified in the corresponding urine cultures.

**Figure 1:**
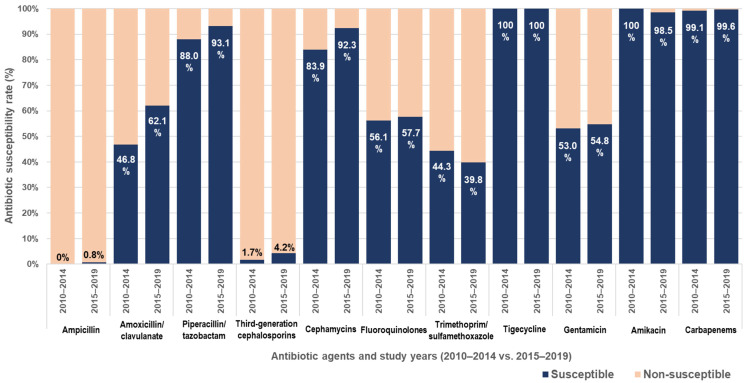
Antibiotic susceptibility rates of ESBL-producing Enterobacterales.

Across the entire study period (2010–2019), antibiotic susceptibility rates were 0.5% for ampicillin, 57.5% for amoxicillin–clavulanate, 91.6% for piperacillin–tazobactam, 3.5% for third-generation cephalosporins, 89.8% for cephamycins, and 99.5% for carbapenems. The corresponding rates for non-β-lactam antibiotics were 54.3% for gentamicin, 98.9% for amikacin, 57.2% for fluoroquinolones, and 41.2% for TMP-SMX. Figure 1 illustrates changes in antibiotic susceptibility rates between the two study periods (2010–2014 and 2015–2019). During the study period, susceptibility rates to amoxicillin–clavulanate (*p* = 0.007) and to cephamycins (*p* = 0.014) increased significantly in the latter five years compared with the first five years (Figure 1).

### 3.2. Antibiotic Effects According to Empirical and Definitive Antibiotics

**Figure 2:**
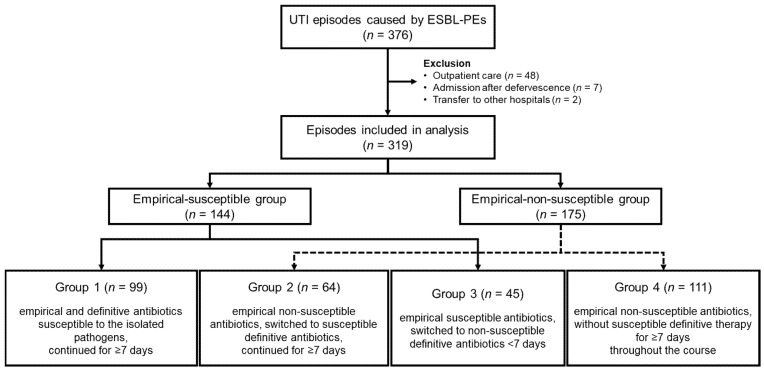
Flowchart for the inclusion, exclusion, and classification of UTI episode groups. UTI: urinary tract infection; ESBL-PEs: extended-spectrum β-lactamase-producing Enterobacterales.

Antibiotic effects were analyzed in 319 episodes from 305 children, including 14 children who experienced two episodes each. Of the total 376 episodes, 48 (12.8%) managed in an outpatient setting, 7 (1.9%) admitted after defervescence, and 2 (0.5%) transferred to other hospitals on the day of admission were excluded, as the exact time of defervescence could not be determined (Figure 2).

Isolated ESBL-PEs were susceptible to empirical antibiotics in 144 (45.1%) episodes, with third-generation cephalosporin plus aminoglycoside administered in 52 (36.1%) of these episodes, amoxicillin–clavulanate plus aminoglycoside in 38 (26.4%), and cephamycin plus aminoglycoside in 25 (17.4%). Carbapenems were administered in only two (1.4%) episodes. Among 175 (54.9%) episodes treated with empirical antibiotics to which the isolated pathogens were non-susceptible, third-generation cephalosporins were administered in 165 (94.3%) episodes. The empirical-susceptible group was significantly older than the empirical-non-susceptible group (*p* < 0.001, Table 2). Although the durations of hospitalization, antibiotic therapy, and fever differed significantly between the two groups (*p* < 0.001), their median values were identical (Table 2). However, fever duration after the initiation of antibiotic therapy, measured in hours, was significantly shorter in the empirical-susceptible group than in the empirical-non-susceptible group (*p* < 0.001, Table 2). The proportions of defervescence within 2 days of hospitalization, defervescence during empirical antibiotic therapy, subsequent urological interventions, and recurrent UTI within 3 months were comparable between the two groups (Table 2).

To evaluate the effects of definitive antibiotics, comparisons were made among Groups 1–4. Susceptible antibiotic therapy for ≥7 days (Groups 1 and 2) was more frequently administered in episodes with accompanying bacteremia (*p* = 0.019, Table 3). Therapy with susceptible antibiotics for ≥7 days was significantly associated with longer durations of hospitalization (*p* < 0.001), antibiotic therapy (*p* = 0.004), and fever after the initiation of antibiotic therapy (*p* < 0.001, Table 3). The proportion of defervescence during empirical antibiotic therapy (*p* = 0.014) was significantly lower in episodes treated with susceptible antibiotics for ≥7 days than in those treated for <7 days (Table 3). However, the recurrence rate of UTI within 3 months showed a significantly increasing trend when non-susceptible antibiotics were administered empirically or when susceptible antibiotics were administered for <7 days (*p* = 0.045, Table 3).

## 4. Discussion

In this study, the susceptibility of isolated ESBL-PEs to empirical antibiotics had no significant impact on clinical improvement and recurrence of febrile UTI. However, when considering the susceptibility to definitive antibiotics and therapy duration with antibiotics to which the isolated pathogens were susceptible, an increasing recurrence rate of ESBL-PE UTI was observed in children who did not receive susceptible antibiotics for ≥7 days.

Empirical therapy with either susceptible or non-susceptible antibiotics showed comparable therapeutic effects and recurrence rates in children with ESBL-PE UTI in previous studies [8,12,13,14,15]. Similarly, adult studies have reported that non-carbapenem antibiotics demonstrated therapeutic effects comparable to those of carbapenems for ESBL-PE UTI [4]. In our study, fever duration after the initiation of antibiotic therapy was slightly longer in the empirical-non-susceptible group than in the empirical-susceptible group, but this difference was unlikely to be clinically meaningful in real-world practice. Therefore, our findings might be considered consistent with earlier reports. Importantly, we focused on the role of definitive antibiotics. Although cephalosporins are regarded as non-susceptible to ESBL-PE, they are the most commonly used empirical agents in children with UTI [17]. However, most previous studies did not evaluate the impact of switching from these non-susceptible agents to susceptible antibiotics [10,18,19]. In our cohort, third-generation cephalosporins were administered empirically in 94.3% of children in the empirical-non-susceptible group, and 63.6% of them completed therapy with these non-susceptible agents. Many children who improved clinically during empirical therapy with non-susceptible antibiotics continued with the same regimen in accordance with general antibiotic practice. However, this approach is inconsistent with the current guideline of the Infectious Diseases Society of America (IDSA), which recommends the use of susceptible antibiotics throughout the treatment course for ESBL-PE infection, except in cases of uncomplicated cystitis, as well as with the AAP guideline, which recommends adjusting antibiotic agents according to susceptibility test results [2,4].

When both the susceptibility to definitive antibiotics and the duration of susceptible antibiotic therapy were considered in this study, the recurrence rate of ESBL-PE UTI tended to increase in children who did not receive susceptible antibiotic therapy for at least 7 days. A previous study on pediatric invasive infections caused by ESBL-PEs, including UTI, also reported a significantly higher recurrence rate in children treated exclusively with third-generation cephalosporins than in those treated with susceptible antibiotics [20]. Some studies on pediatric UTI caused by ESBL-PEs reported comparable recurrence rates between children treated with non-susceptible and susceptible antibiotics; however, each included ≤30 children treated with non-susceptible antibiotics and the duration of therapy with susceptible and non-susceptible antibiotics was not specified [10,18,19]. In our cohort, children who received susceptible antibiotics for ≥7 days had longer durations of fever, hospitalization, and antibiotic therapy compared with those who did not. This likely reflected differences in clinical severity or the timing of clinical response among groups, rather than being caused by antibiotic susceptibility. This trend has also been observed in adult studies evaluating the clinical impact of definitive antibiotics on ESBL-PE UTI [11,21]. Clinically non-severe patients and those showing early clinical response often did not require switching from non-susceptible to susceptible antibiotics, and paradoxically, they experienced more recurrences. Therefore, non-susceptible antibiotics should be replaced with susceptible ones in children with ESBL-PE UTI, and therapy should be continued for ≥7 days, regardless of initial clinical severity or antibiotic response. Considering that carbapenems were used as definitive antibiotics in only 11.0% of episodes in Groups 1 and 2 in this study (Figure S1), non-carbapenem antibiotics may be suitable for complete therapy if the isolated pathogens are susceptible.

Because the overall recurrence rate in our study population was only 5.0%, prolonged hospitalization for parenteral antibiotic therapy may not be cost-effective. Current IDSA and European Society of Clinical Microbiology and Infectious Diseases (ESCMID) guidelines recommend step-down therapy with oral antibiotics after initial parenteral therapy for ESBL-PE UTI [4,22]. In our cohort, 84.0% of isolated ESBL-PE strains were susceptible to orally available antibiotics such as amoxicillin–clavulanate, TMP-SMX, and fluoroquinolones. The IDSA guideline recommends TMP-SMX or fluoroquinolones [4]; however, the susceptibility rate of TMP-SMX was only 41.2% in this study, and fluoroquinolone use remains limited in children. The ESCMID guideline additionally recommends amoxicillin–clavulanate [22]; however, the combined susceptibility rate of amoxicillin–clavulanate and TMP-SMX was 56.9% in this study. Susceptibility rates to amoxicillin–clavulanate and cephamycins increased significantly during the latter half of the study period. Although multiple factors may have influenced these trends, decreased antibiotic pressure from amoxicillin–clavulanate and cephamycins may have contributed to the increased susceptibility [23,24], given the significant rise in the empirical use of third-generation cephalosporins and the corresponding decline in the use of amoxicillin–clavulanate and cephamycins between the two study periods. However, cephamycins are available only in parenteral formulations, and the susceptibility rate of amoxicillin–clavulanate remained approximately 60% even in the latter half of the study period. Further studies are warranted to evaluate the efficacy of cefixime plus amoxicillin–clavulanate combination therapy, which has shown benefit in non-susceptible pathogens in some reports [14,25], as well as the potential role of fluoroquinolones in pediatric ESBL-PE infections.

Although this decade-long study included a larger number of children with ESBL-PE UTI than previous reports, several limitations inherent to its retrospective design could not be avoided. Unmeasured factors influencing antibiotic effects and UTI recurrence might have been missed. Because only 11 episodes of recurrent UTI were identified in this study, the influence of potential confounding factors, such as age, sex, previous UTI episodes, and underlying urogenital abnormalities, on recurrence could not be reliably assessed. Larger nationwide studies including more recurrent events are needed to verify these findings. Antibiotic dose, administration route, and dosing interval could also have affected therapeutic effects. More than 20 combinations of empirical and definitive antibiotics were identified in our cohort; therefore, the effects of each individual antibiotic on clinical improvement and recurrence could not be assessed (Figure S1). We did not evaluate antibiotic effects by differentiating between parenteral and oral therapy. Although oral antibiotic therapy is generally preferred for treating UTI [2], parenteral and oral antibiotics may produce different therapeutic effects. Well-controlled prospective studies using limited antibiotic combinations are required in the future. For the diagnosis of UTI, urine collection by urethral catheterization or suprapubic aspiration is recommended rather than by urine bag [2]. However, in Korea, adhesive urine bags are frequently used in clinical practice. Forcing urethral catheterization or suprapubic aspiration in all children may not be reasonable, given that complications such as hematuria, painful urination, and urinary retention occur in 21% of those undergoing catheterization [26]. One previous study demonstrated identical clinical characteristics and therapeutic responses when children with urine samples collected by urine bags were included versus excluded in the same population [14]. Urine collection using urine bags carries a risk of false-positive UTI diagnosis due to contamination. Concurrent febrile illnesses were identified in approximately 10% of our cohort, but results excluding those children were similar (Table S1). The most concerning long-term renal complications of pediatric UTI are associated with renal scarring [27]; therefore, the association between susceptible antibiotic therapy and renal scarring warrants further investigation. Furthermore, antibiotic resistance rates vary depending on ESBL gene type and *E. coli* genotype [28,29], which were not investigated in this study. Continuous surveillance of ESBL gene and pathogenic genotype distributions in children with UTI would provide valuable information to guide appropriate antibiotic selection. In addition, infection control and prevention strategies aimed at limiting their transmission should be strengthened.

## 5. Conclusions

In conclusion, the susceptibility of ESBL-PEs to empirical antibiotics was not associated with clinical response and recurrence in pediatric UTI. However, definitive therapy with susceptible antibiotics for ≥7 days was significantly associated with a lower rate of UTI recurrence. Even if children with ESBL-PE UTI show an early clinical response during empirical therapy with non-susceptible antibiotics, treatment with susceptible antibiotics for at least 7 days should be considered. These findings support antibiotic stewardship strategies that promote the optimal use of non-carbapenem antibiotics in children with ESBL-PE UTI.

## Figures and Tables

**Table 1:** Clinical characteristics of 376 pediatric UTI episodes caused by ESBL-producing Enterobacterales.

Factor	Number (%)
Year	
2010–2014	115 (30.6)
2015–2019	261 (69.4)
Isolated pathogens	
*Escherichia coli*	366 (97.3)
*Klebsiella pneumoniae*	10 (2.7)
Sex	
Male	249 (66.2)
Female	127 (33.8)
Age, months, median (range)	5 (0–104)
Age group	
<3 months	91 (24.2)
3–5 months	143 (38.0)
6–23 months	101 (26.9)
≥24 months	41 (10.9)
Episodes of UTI	
First	323 (85.9)
Second	36 (9.6)
Third or more	17 (4.5)
Treatment setting	
Hospitalization	328 (87.2)
Outpatient care	48 (12.8)
Previous hospitalization within 3 months	63 (16.8)
Previous antibiotic use within 3 months	77 (20.5)
Urogenital abnormality	56 (14.9)
Abnormal prenatal ultrasonography	9 (2.4)
Vesicoureteral reflux	37 (9.8)
Hydronephrosis	5 (1.3)
Multicystic dysplastic kidney	3 (0.8)
Duplicated collecting system	2 (0.5)
Ureterocele	1 (0.3)
Bladder diverticulum	1 (0.3)
Cloacal anomaly	1 (0.3)
Neurogenic bladder	4 (1.1)
Cryptorchidism	1 (0.3)
Adrenal neuroblastoma	1 (0.3)
Concurrent diagnosis	
Upper respiratory infection	35 (9.3)
Lower respiratory infection	8 (2.1)
Bacteremia	6 (1.6)
Exanthem subitum	4 (1.1)
Febrile seizure	3 (0.8)
Cellulitis	1 (0.3)
Erythema multiforme	1 (0.3)

UTI: urinary tract infection; ESBL: extended-spectrum β-lactamase.

**Table 2:** Comparison of clinical characteristics between empirical-susceptible and empirical-non-susceptible groups.

Factor	Empirical-Susceptible Group (*n* = 144)	Empirical-non-Susceptible Group (*n* = 175)	*p*-Value
Year			<0.001
2010–2014	72 (50.0)	24 (13.7)	
2015–2019	72 (50.0)	151 (86.3)	
Isolated pathogens			1.000
*Escherichia coli*	140 (97.2)	170 (97.1)	
*Klebsiella pneumoniae*	4 (2.8)	5 (2.9)	
Sex			0.295
Male	95 (66.0)	125 (71.4)	
Female	49 (34.0)	50 (28.6)	
Age, months, median (range)	5 (0–101)	4 (0–83)	<0.001
Age group			0.138
<3 months	33 (22.9)	53 (30.3)	
3–5 months	53 (36.8)	71 (40.6)	
6–23 months	40 (27.8)	39 (22.3)	
≥24 months	18 (12.5)	12 (6.9)	
Episodes of UTI			0.892
First	125 (86.8)	154 (88.0)	
Second	14 (9.7)	16 (9.1)	
Third or more	5 (3.5)	5 (2.9)	
Previous hospitalization within 3 months	26 (18.1)	27 (15.4)	0.531
Previous antibiotic use within 3 months	27 (18.8)	32 (18.3)	0.915
Urogenital abnormality	21 (14.6)	24 (13.7)	0.824
Concurrent diagnosis			
Upper respiratory infection	13 (9.0)	16 (9.1)	0.972
Lower respiratory infection	4 (2.8)	4 (2.3)	1.000
Bacteremia ^1^	4 (2.8)	2 (1.2)	0.417
Hospital days, median (range)	7 (3–35)	7 (3–16)	<0.001
Antibiotic duration, days, median (range)			
Total duration	13 (6–34)	13 (2–25)	<0.001
Intravenous antibiotics	6 (2–34)	6 (1–15)	<0.001
Oral antibiotics	7 (0–14)	7 (0–15)	<0.001
Fever duration			
Total, days, median (range)	3 (1–12)	3 (1–12)	<0.001
After initiation of antibiotics, hours, median (range)	11 (0–129)	15 (0–106)	<0.001
Defervescence within 2 days of hospitalization	109 (75.7)	124 (70.9)	0.333
Defervescence during empirical therapy	132 (91.7)	156 (89.1)	0.449
99mTc-DMSA scan abnormality ^2^	58 (81.7)	47 (52.8)	<0.001
Urological intervention	7 (4.9)	8 (4.6)	0.903
Recurrent UTI within 3 months	2 (1.4)	9 (5.1)	0.067

Data are presented as number (%), unless otherwise indicated. UTI: urinary tract infection; 99mTc-DMSA: 99mTc-dimercaptosuccinic acid. ^1^ Blood cultures were performed in 172 episodes of the empirical-non-susceptible group. ^2^ 99mTc-DMSA scans were performed in 71 and 89 episodes in the empirical-susceptible and empirical-non-susceptible groups, respectively.

**Table 3:** Comparison of clinical characteristics among Groups 1–4.

Factor	Group 1 (*n* = 99)	Group 2 (*n* = 64)	Group 3 (*n* = 45)	Group 4 (*n* = 111)	*p*-Value
Year					0.017
2010–2014	40 (40.4)	7 (10.9)	32 (71.1)	17 (15.3)	
2015–2019	59 (59.6)	57 (89.1)	13 (28.9)	94 (84.7)	
Isolated pathogens					0.201
*Escherichia coli*	95 (96.0)	61 (95.3)	45 (100.0)	109 (98.2)	
*Klebsiella pneumoniae*	4 (4.0)	3 (4.7)	0 (0.0)	2 (1.8)	
Sex					0.098
Male	68 (68.7)	38 (59.4)	27 (60.0)	87 (78.4)	
Female	31 (31.3)	26 (40.6)	18 (40.0)	24 (21.6)	
Age, months, median (range)	5 (0–97)	5 (0–80)	5 (1–101)	4 (0–83)	0.072
Age group					0.008
<3 months	21 (21.2)	17 (26.6)	12 (26.7)	36 (32.4)	
3–5 months	37 (37.4)	23 (35.9)	16 (35.6)	48 (43.2)	
6–23 months	29 (29.3)	18 (28.1)	11 (24.4)	21 (18.9)	
≥24 months	12 (12.1)	6 (9.4)	6 (13.3)	6 (5.4)	
Episodes of UTI					0.147
First	84 (84.9)	55 (85.9)	41 (91.1)	99 (89.2)	
Second	11 (11.1)	5 (7.8)	3 (6.7)	11 (9.9)	
Third or more	4 (4.0)	4 (6.3)	1 (2.2)	1 (0.9)	
Previous hospitalization within 3 months	21 (21.2)	10 (15.6)	5 (11.1)	17 (15.3)	0.234
Previous antibiotic use within 3 months	22 (22.2)	11 (17.2)	5 (11.1)	21 (18.9)	0.485
Urogenital abnormality	16 (16.2)	14 (21.9)	5 (11.1)	10 (9.0)	0.059
Concurrent diagnosis					
Upper respiratory infection	9 (9.1)	5 (7.8)	4 (8.9)	11 (9.9)	0.788
Lower respiratory infection	4 (4.0)	1 (1.6)	0 (0.0)	3 (2.7)	0.527
Bacteremia ^1^	4 (4.0)	2 (3.2)	0 (0.0)	0 (0.0)	0.019
Hospital days, median (range)	8 (4–35)	8 (4–16)	6 (3–7)	6 (3–13)	<0.001
Antibiotic duration, days, median (range)					
Total duration	13 (7–34)	14 (9–25)	13 (6–18)	12 (2–18)	<0.001
Intravenous antibiotics	7 (3–34)	7 (3–15)	5 (2–6)	5 (1–12)	<0.001
Oral antibiotics	5 (0–14)	7 (0–15)	8 (0–13)	7 (0–12)	<0.001
Fever duration					
Total, days, median (range)	3 (1–12)	3 (1–10)	3 (1–8)	3 (1–12)	0.336
After initiation of antibiotics, hours, median (range)	12 (0–129)	29 (0–106)	7 (0–85)	7 (0–97)	<0.001
Defervescence within 2 days of hospitalization	73 (73.7)	36 (56.3)	36 (80.0)	88 (79.3)	0.101
Defervescence during empirical therapy	89 (89.9)	49 (71.9)	43 (95.6)	107 (96.4)	0.014
99mTc-DMSA scan abnormality ^2^	46 (90.2)	21 (65.6)	12 (60.0)	26 (45.6)	<0.001
Urological intervention	6 (6.1)	4 (6.3)	1 (2.2)	4 (3.6)	0.301
Recurrent UTI within 3 months	1 (1.0)	2 (3.1)	1 (2.2)	7 (6.3)	0.045

Data are presented as number (%), unless otherwise indicated. UTI: urinary tract infection; 99mTc-DMSA: 99mTc-dimercaptosuccinic acid. ^1^ Blood cultures were performed in 63 episodes in the Group 2 and 109 episodes in the Group 4. ^2^ 99mTc-DMSA scans were performed in 51, 32, 20, and 57 episodes in Groups 1–4, respectively.

## Data Availability

The raw data supporting the conclusions of this article will be made available by the authors on request.

## Supplementary Materials

The following supporting information can be downloaded at: https://www.mdpi.com/article/10.3390/pathogens14111103/s1, Figure S1: Combinations of empirical and definitive antibiotics in UTI episodes caused by ESBL-producing Enterobacterales; Table S1: Comparison of clinical characteristics among Groups 1–4, excluding 41 episodes with other concurrent febrile illnesses.

## Abbreviations

UTI	Urinary tract infection
AAP	American Academy of Pediatrics
ESBL	Extended-spectrum β-lactamase
ESBL-PE	ESBL-producing Enterobacterales
VUR	Vesicoureteral reflux
TMP-SMX	Trimethoprim-sulfamethoxazole
IDSA	Infectious Diseases Society of America
ESCMID	European Society of Clinical Microbiology and Infectious Diseases
