# Supplementary material for: Impact of Empirical and Definitive Antibiotics on Pediatric Febrile Urinary Tract Infection Caused by ESBL-Producing Enterobacterales
Source: Pathogens. 2025 Oct 29;14(11):1103. doi: 10.3390/pathogens14111103 (PMC12655000; doi:10.3390/pathogens14111103)
Supplement: Supplementary file 1 [file pathogens-14-01103-s001.zip › pathogens-3921426-supplementary.pdf]

Table S1. Comparison of clinical characteristics among Groups 1-4, excluding 41 episodes with ot her concurrent febrile illnesses

Factor	Group 1 (*n* = 86)	Group 2 (*n* = 56)	Group 3 (*n* = 41)	Group 4 (*n* = 95)	*p*-value
Year					
2010-2014	36 (41.9)	6 (10.7)	29 (70.7)	12 (12.6)	0.006
2015-2019	50 (58.1)	50 (89.3)	12 (29.3)	83 (87.4)	
Isolated pathogens					
Escherichia coli	82 (95.3)	54 (96.4)	41 (100.0)	94 (98.9)	0.083
Klebsiella pneumoniae	4 (4.7)	2 (3.6)	0 (0.0)	1 (1.1)	
Sex					
Male	59 (68.6)	34 (60.7)	25 (61.0)	75 (78.9)	0.110
Female	27 (31.4)	22 (39.3)	16 (39.0)	20 (21.1)	
Age, months, median (range)	5 (0-97)	5 (0-80)	5 (1-65)	3 (0-83)	0.081
Age group					
<3 months	18 (20.9)	15 (26.8)	12 (29.3)	32 (33.7)	
3–5 months	32 (37.2)	19 (33.9)	15 (36.6)	40 (42.1)	0.006
6–23 months	24 (27.9)	17 (30.4)	10 (24.4)	17 (17.9)	
≥24 months	12 (14.0)	5 (8.9)	4 (9.8)	6 (6.3)	
Episodes of UTI					
First	71 (82.6)	47 (83.9)	38 (92.7)	85 (89.5)	
Second	11 (12.8)	5 (8.9)	2 (4.9)	9 (9.5)	0.068
Third or more	4 (4.7)	4 (7.1)	1 (2.4)	1 (1.1)	
Previous hospitalization within 3 months	18 (20.9)	10 (17.9)	5 (12.2)	14 (14.7)	0.223
Previous antibiotic use within 3 months	19 (22.1)	10 (17.9)	5 (12.2)	17 (17.9)	0.412
Urogenital abnormality	14 (16.3)	14 (25.0)	5 (12.2)	10 (10.5)	0.129
Bacteremia [1]	4 (4.7)	2 (3.6)	0 (0.0)	0 (0.0)	0.019
Hospital days, median (range)	8 (4-24)	8 (4-16)	6 (3-7)	6 (3-13)	<0.001
Antibiotic duration, days, median (range)					
Total duration	13 (7-21)	14 (9-25)	13 (6-18)	12 (7-18)	<0.001
Intravenous antibiotics	7 (3-17)	7 (3-15)	5 (2-6)	5 (1-12)	<0.001
Oral antibiotics	6 (0-14)	7 (0-15)	8 (0-13)	7 (0-12)	<0.001
Fever duration					
Total, days, median (range)	3 (1-12)	3 (1-10)	3 (1-8)	3 (1-12)	0.414
After initiation of antibiotics, hours, median (range)	11 (0-129)	25 (0-106)	7 (0-85)	8 (0-97)	0.022
Defervescence within 2 days of hospitalization	65 (75.6)	35 (62.5)	32 (78.0)	74 (77.9)	0.407
Defervescence during empirical therapy	77 (89.5)	44 (78.6)	39 (95.1)	91 (95.8)	0.034
99mTc-DMSA scan abnormality [2]	42 (91.3)	19 (67.9)	11 (61.1)	23 (45.1)	<0.001
Urological intervention	5 (5.8)	4 (7.1)	1 (2.4)	4 (4.2)	0.468
Recurrent UTI within 3 months	1 (1.2)	2 (3.6)	1 (2.4)	7 (7.4)	0.042

Data are presented as number (%), unless otherwise indicated. UTI: urinary tract infection; 99mTc-DMSA: 99mTc-dimercaptosuccinic acid. [1] Blood cultures were performed in 55 episodes in the Group 2 and 94 episodes in the Group 4. [2] 99mTc-DMSA scans were performed in 46, 28, 18, and 51 episodes in Groups 1-4, respectively.

Figure S1. Combinations of empirical and definitive antibiotics in UTI episodes caused by ESBL-producing Enterobacterales.

Definitive antibiotics \ Empirical antibiotics	AMC	PIT	1st CEF	CXI	3rd CEF	MER	AMP + 3rd CEF	AMP + AG	AMC + CXI	AMC + AG	PIT + AG	CXI + AG	3rd CEF + CXI	3rd CEF + AG	MER + TRS
AMO					1										
AMC	3				12	1	1			12				6	
PIT				1	7									1	
1st CEF			1												
3rd CEF	1	1		10	74		4	1		5		13		24	
MER					12		1	1		4		1		4	
AG										1				1	
TRS				1	7		1	1	1	2		5	1	5	1
CIP												2			
AMP + AG								2							
AMC + 3rd CEF					1					1				1	
AMC + AG	1				6		1			10		1			
PIT + AG										3	1				
CXI + AG				1	2		3								
3rd CEF + AG					35		1					5		6	
3rd CEF + TRS														1	
3rd CEF + CIP														1	
MER + AG										1				1	
AG + TRS										1				1	
3rd CEF + AG + TRS														1	

AMC	Amoxicillin-clavulanate
PIT	Piperacillin-tazobactam
1st CEF	First-generation cephalosporin
CXI	Cefoxitin
3rd CEF	Third-generation cephalosporin
MER	Meropenem
AMP	Ampicillin
AG	Aminoglycoside
TRS	Trimethoprim-sulfamethoxazole
AMO	Amoxicillin
CIP	Ciprofloxacin
